# A cross-sectional study on the prevalence and drug susceptibility pattern of methicillin-resistant *Staphylococcus aureus* isolated from patients in the Buea Health District, Cameroon

**DOI:** 10.11604/pamj.2023.45.28.36860

**Published:** 2023-05-10

**Authors:** Morgan Mokeo Ndedy, Raymond Babila Nyasa, Seraphine Nkie Esemu, Jerome Achah Kfusi, Nene Kaah Keneh, Thomas Njinuwoh Masalla, Lucy Mande Ndip

**Affiliations:** 1 Department of Microbiology and Parasitology, University of Buea, Buea, Cameroon,; 2 Laboratory for Emerging Infectious Diseases, University of Buea, Buea, South West Region, Cameroon

**Keywords:** Methicillin-resistant *Staphylococcus aureus*, wounds, nasopharynx, urine, Buea Health District

## Abstract

**Introduction:**

Staphylococcus aureus, which is part of the normal flora accounts for most acute and chronic infections in humans, and treatment options are greatly limited, when infection is caused by methicillin-resistant Staphylococcus aureus (MRSA). This study was to determine the prevalence and antimicrobial susceptibility pattern of MRSA from clinical samples obtained randomly from patients in Buea Health District.

**Methods:**

a total of 264 wounds, nasopharynx, and urine samples were collected from patients from different hospitals in Buea and transported to the laboratory in the University of Buea, for analysis. Samples were inoculated on mannitol salt agar for S. aureus isolation, characterized morphologically by gram staining and biochemically by catalase, coagulase, and hemolysis tests. Diagnosis of S. aureus was confirmed by molecular identification of the nuc gene. MRSA was identified from S. aureus by oxacillin screening and confirmed by molecular identification of the mecA gene. The data were analyzed using SPSS version 17.0.

**Results:**

S. aureus was isolated from 70 (26.52%) and all were confirmed molecularly by nuc gene amplification. MRSA by oxacillin screening was 36 (13.64%) while MRSA detected by mecA gene amplification was 34 (12.88%). Antimicrobial susceptibility testing revealed 100% resistance to ampicillin, 88.24% to cefixime and 70.59% to ceftriaxone while low resistance was observed to meropenem (11.76%), doxycycline (14.71%), and vancomycin (17.67%).

**Conclusion:**

MRSA isolated from Buea Health District are resistant to ampicillin, cefixime, and ceftriaxone. The antimicrobials (meropenem, doxycycline, and vancomycin) should be used to treat MRSA infections in Buea Health District.

## Introduction

*Staphylococcus aureus* is one of the major organisms causing bloodstream infection worldwide and is very common in nature [1]. It is also part of the normal flora of humans and is commonly present on the skin and nostrils of carriers, which facilitates its transmission by direct contact. This bacterium is a major human pathogen responsible for a wide range of infections, which include; skin, bone, soft tissue, urinary tract infections, pneumonia, health-care-associated bacteremia in community and hospital settings, and other invasive infections. The continuous use of benzylpenicillin (penicillin G) as the drug of choice for treatment of these infections, has led to the emergence of resistant *S. aureus* strains caused by a change in penicillin-binding protein (PBP2a), which is encoded by the *mecA gene*, resulting in strains resistant to penicillins and penicillin-like antibiotics [2]. The emergence of *S. aureus* isolates resistant to vancomycin and other wide range of structurally unrelated antibiotics have elevated MRSA into a multidrug-resistant strain, making it more dangerous than ever in a hospital environment and recently, also in the community [3,4]. A report from the National Nosocomial Infection Surveillance System (NNISS) of the Centers for Disease Control and Prevention (CDCP) (2013-2015) showed that MRSA in India and USA accounts for greater than 60% of *Staphylococcus aureus* isolates which cause nosocomial infections in Intensive Care Units (ICUs) [5].

Several studies have indicated the existence and emergence of MRSA in many health institutions and health districts in Cameroon [2,6] and recent data from Buea, suggest that *Staphylococcus aureus* is the most common bacteria in urinogenital clinical specimens [7]. In 2009 the prevalence of *Staphylococcus aureus* from clinical and environmental samples obtained from Buea was 36.8% and among the isolates, 94.1% and 75.3% were resistant to methicillin and oxacillin respectively and 80% of the isolates were susceptible to vancomycin, 72.9% to ofloxacin and 71.8% to ciprofloxacin [8]. However, the prevalence of MRSA strains and their susceptibility to antimicrobials varies over time. This study aims to determine the current trend in susceptibility of methicillin-resistant *Staphylococcus aureus* infection amongst patients seeking medical care in the Buea Health District.

## Methods

**Study area and population:** the study was carried out in Buea, the capital city of the South West Region of Cameroon. The town is located on the slope of Mount Cameroon and it is bounded by other towns such as Tiko in the south, Muyuka in the east, and Limbe in the west. Buea has an estimated population of 300,000 inhabitants [9]. The population is made up of students, local farmers, business people, government administrators, and civil servants. Two seasons characterize the area, the dry season which starts from November to February, and the rainy season from March to October [10].

**Study design and site:** this was a cross-sectional, hospital-based investigation on the prevalence and drug susceptibility pattern of MRSA isolates in the Buea Health District. Clinical samples were collected between May 12 and July 28 2020 for analysis from three hospitals whose services are most solicited within Buea Health District; Buea Regional Hospital, Mount Mary Hospital, and Solidarity Clinic. Laboratory analysis was carried out in the Laboratory for Emerging Infectious Diseases (LEID), University of Buea, Cameroon.

**Ethical considerations:** participants were educated on the aim and benefit of the study prior to obtaining their informed consent. Assent was obtained from minors, in addition to consent obtained from their parents or guardians. Ethical clearance for this study was obtained from the University of Buea, Faculty of Health Sciences- Institutional Review Board, reference number 2020/1225-07/UB/SG/IRB/FHS, and administrative clearance from Ministry of Public Health Regional Delegation for South West, reference number R11/MINSANTE/SWR/RDPH/PS/703/729.

**Sampling strategy:** simple random and purposive sampling methods were used to select health facilities from the different categories of health care within the Buea Health District and the number of patients recruited from each health facility was proportionate to the patient population of the health facility. The Buea Regional Hospital was purposively selected since it is the only secondary-level referral health facility in the Buea Health District, while the Mount Mary Hospital was randomly selected from the two confessional hospitals of the district (Seventh Day Adventist Hospital and Mount Mary Hospital). The Solidarity Clinic was selected randomly from all private health facilities. A consecutive and non-probabilistic method was used to enroll participants to obtain either a wound swab or nasopharyngeal swab or urine sample from each patient of either sex of all age groups, resident in the Buea Health District. Urine samples were those of patients presenting urine for analysis in the laboratory; wound swabs from patients with old wounds and nasal swabs from admitted patients and patients soliciting for laboratory examination.

**Sample size:** the sample size was calculated using Fisher´s formula [11].


$$n=\frac{z^{2}P\left(1-P\right)}{d^{2}}$$

Where: n=sample size; Z=statistics for level of confidence = 1.96; d=degree of precision = 0.05; P=prevalence of the disease under investigation=13.16%. P was obtained from the prevalence of MRSA from clinical samples in Laquintinie Hospital Douala, Cameroon, a nearby town to Buea, which was 13.16% [6]. P=13.16/100 = 0.132.


$$n=\frac{1.96^{2}x0.132\left(1-0.132\right)}{0.05^{2}}$$

n=176 (minimum sample size required). However, a total of 264 samples were collected; 109 from Buea Regional Hospital, 130 from Mount Mary Hospitals, and 25 from Solidarity Clinic. The 264 samples were composed of 107 urine samples, 50 wound swabs, and 107 nasal swabs.

**Figure 1:**
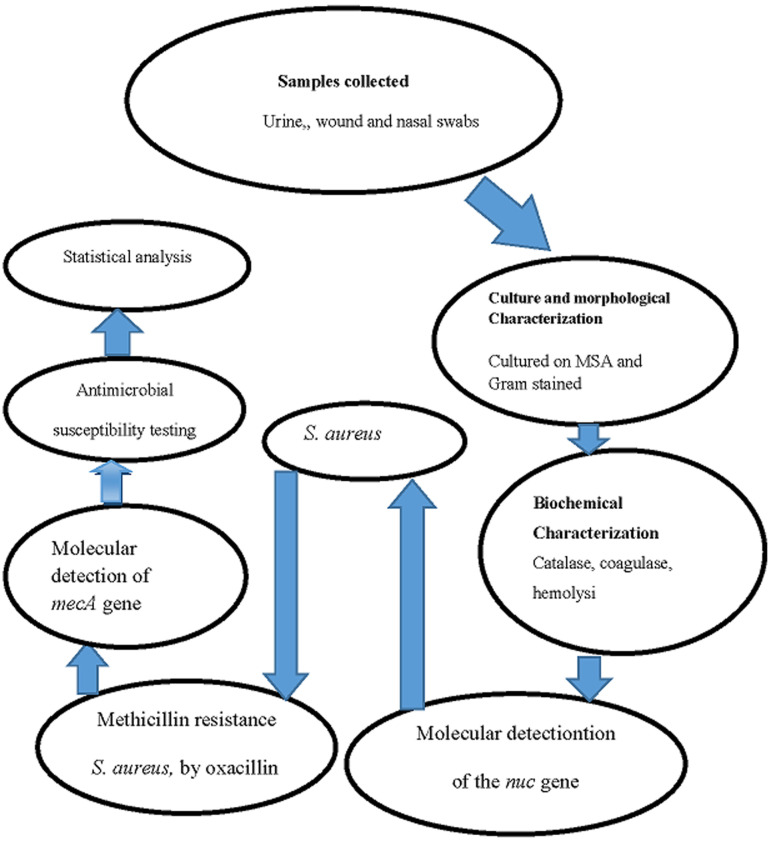
sample collection and processing algorithm

**Laboratory analysis:** the workflow from sample collection to data processing is shown in Figure 1. Samples were collected and inoculated on mannitol salt agar, followed by gram staining of yellow colonies to observe for gram-positive cocci in clusters. Colonies presenting these characteristics were purified on nutrient agar and characterized biochemically using catalase, coagulase, and hemolysis tests. Isolates that were positive for catalase, and coagulase and showed beta hemolysis on blood agar were inoculated into 800μL of nutrient broth in Eppendorf tubes, vortexed and incubated at 37°C for 24h. The tubes were then centrifuged at 8000rpm for 5min, 500 to 600μL of the supernatant was dispensed, and 800μL of 50% glycerol was added and the mixture vortexed briefly for 10secs and stored at -20°C for further analysis (Figure 1).

**Molecular identification of *Staphylococcus aureus*:** molecular identification of *S. aureus* targeted the *nuc gene* using singleplex. To achieve this, stored isolates at -20°C were brought to room temperature, inoculated on nutrient agar, and incubated at 37°C for 24h to obtain colonies for deoxyribonucleic acid (DNA) extraction. Bacterial DNA was extracted using the boiling method [12]. Five to ten colonies were suspended in 150μL of phosphate-buffered saline and heated in a water bath at 100°C for 15 min, chilled on ice for 15 min and allowed to thaw at room temperature for 15 min. The freeze-thaw step was repeated and lysed cells were centrifuged at 14000 rpm for 5 min to obtain the supernatant (bacterial DNA) as reported elsewhere [13]. Presence of bacterial DNA in the supernatant was ascertained by agarose gel electrophoresis, stained with Ethidium bromide to observe for bands under ultraviolet light. Polymerase chain reaction (PCR) reaction was set up using 6.5μL of RNase-free water, 5μL supernatant containing bacterial DNA as template, 12.5μL of master mix, 0.5μL each of reverse (GCG ATT GAT GGT GAT ACG GTT) and forward (AGC CAA GCC TTG ACG AAC TAA AGC) primer (Inqababiotec, SA.), to achieve a final volume of 25μL and a final primer concentration of 10μM, as has been used elsewhere [14]. Following optimization, the 280bp fragment of *nuc* gene was amplified using a thermal cycler (MyCycler™ Thermal Cycler BIORAD, USA) at an initial denaturation of 95°C for 10min, followed by 40 cycles of 1sec denaturation at 94°C for 1min, annealing at 54°C for 30 sec and extension at 72°C for 1min, with a final extension at 72°C for 7 min. The PCR products were electrophoresed at 90volt for 1h in a 1.5% agarose gel, stained with ethidium bromide, visualized under ultraviolet light and photographed using Gel Documentation-XR (BIORAD, Hercules, CA).

**Identification of methicillin-resistant *Staphylococcus aureus*:** colonies of confirmed *Staphylococcus aureus* isolates were screened for oxacillin resistance by the Kirby-Bauer disc-diffusion technique. They were suspended in 0.9% NaCl, to achieve an optical density equivalent to 0.5 McFarland barium sulfate standard (1.5 x 10^8^CFU/ml), as used elsewhere [15], inoculated on Mueller Hinton agar by spread plate technique, followed by the introduction of oxacillin discs and incubation at 37°C for 24 hours. Clear zones of ≥13 mm around the discs revealed susceptibility while ≤10 mm or no clear zones revealed resistance to oxacillin.

Molecular identification of *mecA* gene in oxacillin-resistant isolate was used to confirm methicillin-resistant *S. aureus* by PCR. The reaction was composed of 12.5μL of the master mix, 6.5μL of RNase-free water, 5μL the DNA template, 0.5μL of reverse (AGT TCG CAGTTA CCG GAT TTGC) and 0.5μL of forward (AAA ATC GAT AAA GGT TGGC) primers as reported elsewhere [16], to achieve a final volume of 25μL. Following optimization, the reaction was carried out at an initial denaturation at 95°C for 5min, followed by second denaturation at 94°C for 1min, annealing at 50°C for 1min and extension at 72°C for 1min with a final extension at 72°C for 5min after 35 cycles. The products were run on a 1.5% agarose gel alongside with a molecular ladder and stained with Ethidium bromide to confirm the position of the 533bp *mecA* gene.

**Determination of antimicrobial susceptibility pattern of MRSA:** Kirby Bauer disc diffusion method was used to determine the anti-biogram of MRSA using vancomycin (30μg), ampicillin (10μg), doxycycline (30μg), ofloxacin (5μg), azithromycin (15μg), amikacin (30μg), clindamycin (2μg), cefixime (5μg), ceftriaxone (30μg) and meropenem (10μg) discs. In essence, approximately 1.5x10^8^CFU/mL cell density were used to inoculate Muller Hinton agar plate and antibiotic discs were placed at least 24 mm apart and incubated at 35°C for 24h. The diameters of antibiotic inhibition zone were measured and the results obtained were used to classify isolates as being resistant, intermediate resistant or susceptible to a particular antibiotic using standard reference values by the Clinical and Laboratory Standards Institute (CLSI, 2013) [17].

**Statistical analysis:** data were analyzed using Microsoft Excel 2016 and the statistical software SPSS version 17.0. Descriptive statistics was used to obtain prevalence of *S. aureus* and MRSA, while the Chi-square test was used to investigate the association between *S. aureus*, and MRSA with demographic factors. Statistical significance was set at p<0.05. The percentage sensitivity of MRSA isolates to the different antibiotics were presented on bar charts.

## Results

**Socio-demographic data of patients:** out of the 264 samples collected from patients most (177/264) were females (Table 1). Based on age, majority of participants were between the ages of 21-40 years (155/264). The majority (130/264) of the patients were from Mount Mary Hospital and urine samples constituted 107, nasal swabs were 107, and 50 wound samples.

**Table 1:** socio-demographic characteristics of study participants

Characteristics	Frequency	Percentage (%)
**Age (years)**		
≤20	41	15.53
21-40	155	58.71
41-60	45	17.04
>60	23	8.71
Total	264	100
**Gender**		
Male	87	32.95
Female	177	67.05
Total	264	100
**Sample type**		
Urine	107	40.53
Wound	50	18.94
Nasal swab	107	40.53
Total	264	100
**Hospitals**		
Buea Regional Hospital	109	41.29
Mount Mary Hospital	130	49.24
Solidarity Health Clinic	25	9.47
Total	264	100

**Isolation and identification of *Staphylococcus aureus*:** from a total of 264 samples inoculated on mannitol salt agar, 172 fermented mannitol appeared as yellow colonies with yellow zones surrounding the colonies on the agar plate. Out of the 172 samples that fermented mannitol, 164 of the samples were gram-positive cocci occurring in clusters, which were purified on nutrient agar. The purified colonies were observed to be catalase positive (164), of which 156 were coagulase-positive, and DNA was isolated from 96 samples which showed hemolysis on blood agar, from the 156 coagulase-positive samples. The DNA extracted from the 96 samples was separated by electrophoresis and stained with ethidium bromide for visualization. Extracted DNA was also used for *nuc* gene detection by PCR. Seventy of 96 isolates were positive for the *nuc* gene with a molecular weight of 280bp.

**Occurrence of *Staphylococcus aureus* isolate with respect to age, gender, sample type, and hospital:** *Staphylococcus aureus* infection did not differ with gender (26.55% in females and 26.44% in males) and aged group, with all age groups having a prevalence of 26% (Table 2). The majority (36.45%) of the urine samples were contaminated with *S. aureus* while 4 (8.00%) of the isolates were obtained from wound samples and 25.23% of the nasal swabs were contaminated with *S. aureus* and this difference was significant (p=0.001). Most (38 (29.23%) of the *S. aureus* isolates were isolated from patients attending the Mount Mary Hospital compared to other hospitals (p=0.25).

**Table 2:** distribution of *S. aureus* isolates with respect to age, gender type of sample and the hospital

Characteristics	No. of examined	No. of positive (%)	P-value
**Age (years)**			
≤20	41	11 (26.83)	0.815
21-40	155	41 (26.45)	
41-60	45	12 (26.67)	
>60	23	6 (26.09)	
Total	264	70 (26.52)	
**Gender**			
Male	87	23 (26.44)	0.860
Female	177	47 (26.55)	
Total	264	70 (26.52)	
**Sample type**			
Urine	107	39 (36.45)	0.001
Wound	50	4 (8.00%)	
Nasal swab	107	27 (25.23)	
Total	264	70 (26.52)	
**Hospitals**			
Buea Regional Hospital	109	21 (19.27)	0.025
Mount Mary Hospital	130	38 (29.23)	
Solidarity Health Clinic	25	11 (44.00)	
Total	264	70 (26.52)	

**Phenotypic and molecular identification of MRSA by oxacillin screening:** all the 70 isolates in which the *nuc* gene was amplified were screened for methicillin resistance using oxacillin. Clear zones around the discs ≥13 mm revealed susceptibility while little or no clear zones (≤10 mm) revealed resistance and values between 11-12mm were judged as intermediate. Generally, out of the 264 clinical samples analyzed, 194 samples were negative for *nuc* gene while 70 (26.52%) of the isolates had the *nuc* gene. Thirty-four (12.88%) of the *S. aureus* were oxacillin susceptible (OSSA) while 36 (13.64%) were oxacillin-resistant *Staphylococcus aureus* (ORSA). Of the 36 samples, which were oxacillin resistant *MecA* gene was amplified in 34 (12.88%) of the isolates.

**Occurrence of methicillin-resistant *S. aureus* based on *mecA* gene amplification and demographic factors:** majority of the MRSA (6 (54.55%)) were isolated from individuals of ages ≤20 years, although there was no significant difference between age groups (p=0.77) as shown in Table 3. Male gender (60.87%), wound samples (75.00%) had the highest prevalence of MRSA, but were not significantly different from the others, while there was a significant difference in the occurrence of *S. aureus* amongst hospitals (p=0.00) with Solidarity Hospital (72.73%) having the highest occurrence of MRSA. Most of the MRSA were isolated from males (14) and urine samples (20) (Table 3).

**Table 3:** distribution of methicillin-resistant *Staphylococcus aureus* by *mecA* gene amplification with respect to age, gender, sample type and hospital

Characteristics	No of examined	No of positive (%)	P-value
**Age (years)**			
≤20	11	6 (54.55)	0.770
21-40	41	20 (48.78)	
41-60	12	5 (41.67)	
>60	6	3 (50.00)	
Total	70	34 (48.57)	
**Gender**			
Male	23	14 (60.87)	0.834
Female	47	20 (42.55)	
Total	70	34 (48.57)	
**Sample type**			
Urine	39	20 (51.28)	0.490
Wound	4	3 (75.00)	
Nasal swab	27	11 (40.74)	
Total	70	34 (48.57)	
**Hospitals**			
Buea Regional Hospital	21	6 (28.57)	0.000
Mount Mary Hospital	38	20 (52.63)	
Solidarity Health Clinic	11	8 (72.73)	
Total	70	34 (48.57)	

**Figure 2:**
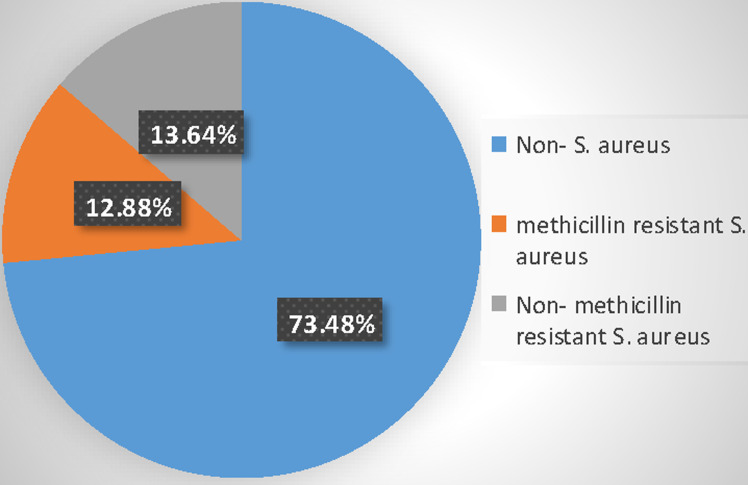
prevalence of *mecA* positive methicillin-resistant *Staphylococcus aureus*

**Prevalence of MRSA by *mecA* gene amplification:** out of 264 clinical samples analyzed, *S. aureus* was isolated from 70 (26.52%) and MRSA with *mecA* gene was amplified from 34 (12.88%) but the gene was absent in 36 (13.64%) of the isolates (Figure 2).

**Antimicrobial susceptibility pattern of MRSA strains to different antimicrobial agents:** the antimicrobial susceptibility test revealed that 34 (100%) were resistant to ampicillin, 30 (88.24%) were resistant to cefixime, 24 (70.59%) were resistant to ceftriaxone and 20 (58.82%) were resistant to azithromycin (Table 4). Also, 17 (50.00%) were resistant to clindamycin, 14 (41.18%) were resistant to ofloxacin, 8 (23.53%) were resistant to amikacin, 6 (17.65%) of the isolates were resistant to vancomycin, and 4 (11.76%) were resistant to meropenem. Thirty of the isolates (88.24%) were susceptible to meropenem, 28 (82.35%) were susceptible to vancomycin, 25 (73.53%) were susceptible to doxycycline, 23 (67.65%) were susceptible to amikacin and 17 (50.0%) were susceptible ofloxacin while 4 (11.79%) were susceptible to ceftriaxone and cefixime. Likewise, 8 (23.53%) were susceptible to azithromycin and 11 (32.35%) were susceptible to clindamycin. None of the isolates were intermediate to vancomycin, ampicillin, cefixime, and meropenem while 6 (17.65%) were intermediate to ceftriaxone, azithromycin, and clindamycin. Furthermore, 3 (8.82%) were intermediate to ofloxacin and amikacin and lastly, 4 (11.76%) were susceptible to doxycycline (Table 4).

**Table 4:** antimicrobial susceptibility pattern of methicillin-resistant *Staphylococcus aureus* strains

Antibiotics	Resistant (%)	Intermediate (%)	Sensitive (%)
Vancomycin	6 (17.65)	0 (0.00)	28 (82.35)
Doxycycline	5 (14.71)	4 (11.76)	25 (73.53)
Ofloxacin	14 (41.18)	3 (8.82)	17 (50.00)
Ampicillin	34 (100)	0 (0.00)	0 (0.00)
Cefixime	30 (88.24)	0 (0.00)	4 (11.76)
Clindamycin	17 (50.00)	6 (17.65)	11 (32.35)
Meropenem	4 (11.76)	0 (0.00)	30 (88.24)
Amikacin	8 (23.53)	3 (8.82)	23 (67.65)
Azithromycin	20 (58.82)	6 (17.65)	8 (23.53)
Ceftriaxone	24 (70.59)	6 (17.65)	4 (11.76)

**Figure 3:**
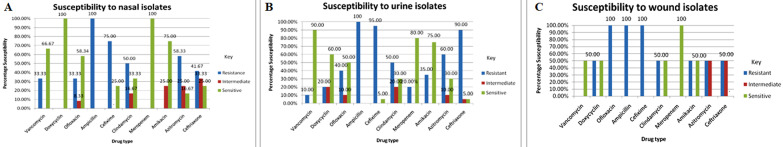
(A,B,C) antimicrobial susceptibility pattern of methicillin-resistant *Staphylococcus aureus* strains isolates from the different sample types

**Antimicrobial susceptibility pattern of MRSA isolates from the different sample types:** out of the 34 MRSA isolates with *mecA* gene, 11 (4.17%) were from nasal swabs, 20 (7.58%) from urine samples and 3 (1.14%) from wound swabs. A total of 11 (100%) of the isolates from nasal swabs were sensitive to meropenem, 11 (100%) were sensitive to doxycycline, 9 (81.82%) were sensitive to amikacin, 8 (72.73%) were sensitive to vancomycin and 7 (63.64%) were sensitive to ofloxacin (Figure 3A). The least sensitive of the isolates were; ceftriaxone 3 (27.27%), azithromycin 2 (18.18%), and cefixime 3 (27.27%). Also, 11 (100%) of the nasal isolates were resistant to ampicillin, 9 (81.82%) were resistant to cefixime and 7 (63.64%) were resistant to azithromycin. For urine samples, 18 (90.00%) were sensitive to vancomycin, 12 (60.00%) were sensitive doxycycline, 10 (50.0%) were sensitive ofloxacin, 16 (80.00%) were sensitive meropenem and 13 (75.00%) were sensitive amikacin (Figure 3B). All the isolates from the urine; 20 (100%) were resistant to ampicillin and 19 (95.00%) were resistant to cefixime. Of all the isolates from wound samples; 3 (100%) of the isolates were sensitive to vancomycin and meropenem while 3 (100%) were resistant to ampicillin, ofloxacin, and cefixime (Figure 3C).

## Discussion

Based on morphological and biochemical characteristics, 36.36% of the total samples analyzed were *Staphylococcus aureus*, however, only 26.52% were confirmed by molecular techniques for the presence of the *nuc* gene. This value is similar to the 36% prevalence of *S. aureus* reported in Zaria, Nigeria [18], but differs from 57.5% prevalence observed in Anbar, Bangladesh [19]. In this study, the overall prevalence of MRSA was 12.88% while that of methicillin-susceptible *Staphylococcus aureus* (MSSA) was 13.64%. This is similar to 13.16% reported in Douala [6], but higher than 5.26% reported in Peshawar, Pakistan [20]. These variations in the prevalence of MRSA could be as a result of differences in environmental factors, methodology employed, number of samples used, or other practices used in the control of the infections.

Although there was no significant difference in the isolation of MRSA with respect to age, gender, and sample types, except for the different hospitals, MRSA was isolated more from males (60.87%) than females (42.55%). This result is similar to previous studies in Douala, Cameroon, where a higher prevalence was recorded in males 53.33% than in females 46.67% [6]. However, the difference was not significant. The majority (48.78%) of the MRSA was isolated from participants aged 21-40 years old and this is in agreement with a similar study carried out in Yaoundé, the capital of Cameroon where the age group 21-40 years old had the highest prevalence of 56.2% [2], and in Douala, the economic capital of Cameroon, they also had the highest prevalence of 66.67% [6]. The highest occurrence of MRSA was observed in urine samples (51.28%) compared to the other samples. This high prevalence of MRSA in urine has also been reported in Nigeria [21]. A high prevalence of the MRSA strains was observed in Solidarity Clinic (72.73%) and Mount Mary Hospital (52.63%) than in the Buea Regional Hospital (28.57%) and the differences were significant. It has been observed that the percentage of MRSA strains has increased worldwide in the last two decades and the percentage varies markedly across different countries and among hospitals of a particular country [2]. Improper infection prevention practices in the different hospital set up, random use of antibiotics; hospitalization in intensive care unit contribute to the emergence and spread of MRSA [2]. These factors as well as the differences in the study population may explain these variations.

Penicillin showed the highest level of resistance (100%) against MRSA isolates. This is similar to a previous study in Douala, the economic capital of Cameroon where high levels of resistance were also observed against penicillins (80-100%) [6]. All MRSA isolates with *mecA* genes that were resistant to oxacillin, were also resistant to ampicillin 34 (100%). This result is similar to a study in Zaria, Nigeria where 100% of the isolates were resistant to ampicillin [22]. This is obvious since MRSA are bacteria that have acquired the gene for beta-lactamase production; an enzyme that breaks the beta-lactam ring in penicillins [6]. However, resistance against meropenem (11.76%), doxycycline (14.71%), and vancomycin (17.67%) were the least. This is closely in line with a similar study in Kabul, Afghanistan with 0.00% resistance to vancomycin, 16.4% resistance to meropenems, 8.6% resistance to doxycycline [23]. Thus, these are the recommended antibiotics for the treatment of MRSA in Buea Health District and may also suggest that, these antibiotics are not commonly overused and abused in the health district. In this study, the highest sensitivity was observed against meropenem (88.24%), which is higher than the 34.2% observed in a study conducted in Houston, USA [24].

Based on the antibiotic susceptibility pattern of MRSA from different anatomical sites to the different antibiotics, this study showed that 100% of the nasal isolates were sensitive to meropenem and doxycycline and 81.82% of the isolates were sensitive to amikacin. For the urine sample, 90% were sensitive to vancomycin, 60% were sensitive to doxycycline while 80% were sensitive to meropenem, and 75% were sensitive to amikacin. Isolates from wound samples showed 100% sensitivity to meropenem and vancomycin. Generally, though the isolates were more sensitive to meropenem, vancomycin, and doxycycline, the susceptibility of isolates from different sites showed some differences, indicating that particular drugs are preferred for the treatment of infection in specific sites.

## Conclusion

A high prevalence of MRSA (12.88%) was observed in Buea Health District, which were resistant to ampicillin (100%), cefixime (88.24), ceftriaxone (70.59%) but sensitive to meropenem (88.25%), vancomycin (82.35%) and doxycycline (73.53%).

### What is known about this topic

- S. aureus which are part of the normal flora can cause acute and chronic infections in humans;
- S. aureus are known to colonize the upper respiratory tract, urinogenital tracts, and wounds;
- Methicillin-resistant Staphylococcus aureus poses a greater health threat with limited options for treatment.

### What this study adds

- A high prevalence of 12.9% colonization with MRSA amongst patients seeking for medical attention in the Buea Health District;
- MRSA isolated from the Buea Health District are resistant to ampicillin, cefixime and ceftriaxone;
- MRSA isolated from Buea Health District is sensitive to meropenems, doxycycline, and vancomycin.
